# Uncovering the Mast Cell Response to *Mycobacterium tuberculosis*

**DOI:** 10.3389/fimmu.2022.886044

**Published:** 2022-06-02

**Authors:** Ivonne Torres-Atencio, Ariadne Campble, Amador Goodridge, Margarita Martin

**Affiliations:** ^1^ Departamento de Farmacología, Facultad de Medicina, Universidad de Panamá, Panama, Panama; ^2^ Tuberculosis Biomarker Research Unit, Centro de Biología Molecular y Celular de Enfermedades (CBCME) – Instituto de Investigaciones Científicas y Servicios de Alta Tecnología (INDICASAT-AIP), Ciudad Del Saber, Panama; ^3^ Biochemistry Unit, Biomedicine Department, Faculty of Medicine, University of Barcelona, Barcelona, Spain; ^4^ Laboratory of Clinical and Experimental Respiratory Immunoallergy, August Pi i Sunyer Biomedical Research Institute (IDIBAPS), Barcelona, Spain

**Keywords:** tuberculosis, mast cells, disease control, histamine, co-infection, biomarker, anti-TB therapy, disease progression

## Abstract

The immunologic mechanisms that contribute to the response to *Mycobacterium tuberculosis* infection still represent a challenge in the clinical management and scientific understanding of tuberculosis disease. In this scenario, the role of the different cells involved in the host response, either in terms of innate or adaptive immunity, remains key for defeating this disease. Among this coordinated cell response, mast cells remain key for defeating tuberculosis infection and disease. Together with its effector’s molecules, membrane receptors as well as its anatomical locations, mast cells play a crucial role in the establishment and perpetuation of the inflammatory response that leads to the generation of the granuloma during tuberculosis. This review highlights the current evidences that support the notion of mast cells as key link to reinforce the advancements in tuberculosis diagnosis, disease progression, and novel therapeutic strategies. Special focus on mast cells capacity for the modulation of the inflammatory response among patients suffering multidrug resistant tuberculosis or in co-infections such as current COVID-19 pandemic.

## Mini Review

*Mycobacterium tuberculosis* (MTB) is the bacterium that causes tuberculosis (TB), which surpasses Human Immunodeficiency Virus (HIV) as the leading cause of death by an infectious agent. According to the World Health Organization (WHO), in 2019, approximately 10 million people became ill with active tuberculosis, and 1.2 million died from this disease (1). It is estimated that one third of the world population is infected with this bacterium but remains asymptomatic. Before the coronavirus (COVID-19) pandemic, TB was the leading cause of death from a single infectious agent (1).

Despite advances made over the past 20 years in understanding the mechanisms that contribute to protective immunity against MTB, there are still major gaps in scientific and clinical knowledge regarding TB. One of these gaps is TB latency, specifically the mechanisms that allow MTB to remain clinically latent and how the infection is reactivated, and disease development occurs. Another crucial point that remains unclear is the individual roles of the different immune cells participating in the host response to the bacilli. The existence of multi-drug resistant strains adds yet another layer of complexity to understanding interactions between the bacilli and genetically distinct hosts. These represent the main obstacles to developing treatments directed at latent infections as a means to prevent disease (2).

Under the current COVID-19 pandemic conditions, another cause for concern is possible co-infection with MTB and SARS-CoV-2 virus. In addition to co-infected patients presenting more severe respiratory symptoms, the cellular mediators released during acute COVID-19 infection increase the risk of latent tuberculosis reactivation (3). Mast cells are among these cellular mediators and play a role in mast cell activation syndrome (MCAS) (4). While MCAS could be exacerbated with multiple pathogen infections, no major MCAS among patients with triple infections of TB, HIV, and SARS-CoV-2 has been noticed (5, 6). Rivas et al. reported two unusual cases of patients with triple infections of SARS-CoV-2, MTB, and HIV. Following treatment with supplemental oxygen and initiation of antituberculosis and antiretroviral therapies, both patients demonstrated clinical improvement and recovery from COVID-19. Thus, the effect of multiple infection on mast cell response requires a deeper revision to denote differences between clinical observations and the cellular and molecular level of MCAS.

Host resistance to MTB infection requires a coordinated effort between cells of the innate and adaptive immune systems (7). Various lung immune cell populations respond to MTB, and while their functions offer host protection, they can also result in potentially damaging inflammation. For example, although CD4+ T cells are mainly responsible for containing the infection through specific antigen recognition, these T cells are also major contributors to disease at different stages of infection (8). Macrophages, neutrophils, dendritic cells, NK, and B lymphocytes have also been shown to play an important role in the MTB infection response (9). Thus, the participation of both adaptative and innate immune branches remains key for defense against tuberculosis.

While the roles of these immune cells have been determined, the role of mast cells (MC) remains unclear. This minireview aims to consolidate the knowledge available on the host’s MC response to MTB. Special focus will be directed to the cellular and clinical aspects related to this interaction.

## MCs Function

MCs’ versatility results from several elements that characterize their immune protection skills. First, MCs store a variety of mediators in their granules and express a diverse population of receptors on the cell membrane. Second, MCs are strategically distributed throughout the body in sites whose function complements the immune system. Specifically, MCs are found in skin and gastrointestinal and respiratory tissues and mucous membranes (10). The diversity of MC receptors, their signaling pathways and products make MCs central effector cells in the pathogenesis of many respiratory diseases, particularly diseases with underlying inflammatory and allergic pathologies (11).

In addition to the role mast cells play in allergenic processes, increasing evidence points to the participation of these cells in other pathologies ranging from autoimmune disorders, infectious diseases to cancer. In these processes, MCs have deleterious effects. Understanding how MCs cause damage as well as finding strategies to limit these effects is an important area of research (10).

MCs are further classified into two subtypes according to their protease content. Those found in skin are mostly chymase-tryptase positive (MC_TC_), while those located in mucosa, such as bronchial epithelium, are tryptase positive (MC_T_). In addition to the MC subtype, the increase in the number of MCs is also important in inflammatory processes (12). The major activation pathway is through the FcϵRI and its interaction with IgE. Other MC receptors have a sentinel response to lipopolysaccharides or peptidoglycans generated by bacteria. For example, upon activation, Toll-Like Receptors (TLRs) induce the synthesis and up-regulation of cytokines including GM-CSF, IL-6, IL-8, and IL-10 (13). The precise cytokine profile depends on the activation route within the mast cell. To facilitate this interaction, MCs are located at the host-environment interface, proximal to both blood and lymphatic vessels. In addition, MCs are present along nerve fibers and close to tissue-resident immune cells (i.e. dendritic cells) (14).

Advancements in our understanding of MC biology have identified an abundance of checkpoints for potential therapeutic intervention. For example, the introduction of antihistamines more than 80 years ago led to the discovery of the different histamine receptors, one of the major inflammatory mediators stored in MCs (15). H1-antihistamines represent the cornerstone of treatment in patients with pruritus, allergic rhinitis, or allergic conjunctivitis, and this receptor is abundantly expressed on MCs (15).

## MC Antimicrobial Activity

MCs demonstrate antimicrobial activity against bacteria, fungi, and viruses (14). These functions are executed following pathogen recognition *via* pattern recognition receptors (PRRs), and Fc receptors. At the beginning of an infection, MCs signal the pathogen’s presence to innate and adaptive immune cells present near the infection site or distally in the draining lymph nodes (14). Mouse models of infectious pathogens have demonstrated increased pathogen clearance in animals with sufficient MCs compared to MC-deficient mice (16).

MCs have unique features that make their functions crucial for host defense. First, they are preponderant in the skin and mucosal sites, which is where most pathogens initiate infection, and they are among the first cells to encounter pathogens along with other innate immune cells, such as dendritic cells and macrophages. Second, MCs can recognize three targets: the pathogens directly, host molecules that opsonize pathogens, and/or signals released by infected cells in the vicinity. Third, after activation, MCs release inflammatory mediators and antimicrobial peptides that contribute to immune cell recruitment and the clearance of the invading pathogen (14).

Moreover, MCs can both initiate and sustain the inflammatory response due to *de novo* synthesis of mediators and their ability to undergo multiple regranulation-degranulation cycles. One remarkable feature that distinguishes MCs from all other granulated cell types (e.g. neutrophils) is that cellular degranulation is not automatically associated with cell death. Instead, early studies suggest that MCs not only survive this process but possess the ability to recover, replenish their granules and participate in another round of degranulation (17). This special ability of regranulation makes sense considering the longevity and high time and energy investment in terms of weeklong development and maturation of MCs (18). On the other hand the ability of MCs to independently phagocytize and kill bacteria has also been discussed and remains controversial (19). In recent years, an additional antimicrobial mechanism, known as mast extracellular traps (MECT), has been proposed (20).

## Microbial Signals That Activate MCs: PAMPS and DAMPS

MCs activation mechanisms differ according to the pathogen. Depending on the stimuli perceived by MCs, they release a panel of inflammatory mediators for the type of pathogen detected (21). For example, in one study, MCs secreted proinflammatory cytokines when challenged by respiratory syncytial virus, they secreted anti-viral type 1 IFN (22). This differential response results from the characteristics of the MC surface receptors that are engaged and the signals released by those receptors.

MCs express a wide array of pattern recognition receptors (PRRs). The PRRs are a class of germ line-encoded receptors that recognize both extra- and intracellular pathogens as well as pathogen-derived products and pathogen-associated molecular patterns (PAMPS) (23). Among these PRRs, TLR expression in MCs from various origins has been well documented. Human MCs have been shown to express TLR1, TLR2, TLR3, TLR4, TLR5, TLR6, TLR7, TLR8, and TLR9 at different levels depending on the literature consulted (24–26).

The best-characterized receptors are TLR2, TLR4, and TLR5, which recognize the following bacterial products: peptidoglycan, lipopolysaccharide (LPS), and Flagelin, respectively (14). Cytokine and chemokine production and secretion are the common functional outcomes of TLR signaling in MCs, but this signaling can also induce calcium influx and degranulation (e.g. peptidoglycan stimulation through TLR2) (27).

MTB can be recognized by MC PRRs, as is the case with TLR2 (28). In addition, MCs can respond to danger signals (DAMPS), such as IL-33, released by neighboring infected host cells, which activates proinflammatory cytokine secretion by MCs to resolve infection (29). Previous exposure to pathogens/TLR-ligands enhances IgE-dependent release of several inflammatory mediators in MCs, which suggests that infections might magnify the severity of allergic reactions (30). In addition to the PPRs, other molecules, such as the glycosylphosphatidylinositol-anchored proteins (GPI) receptor CD48, can bind pathogens and trigger MC degranulation and microbe uptake (31). Complement receptors (CR3, CR4, C3aR, and C5aR) and immunoglobulin receptors (FcϵRI, FcγRI, FcγRII, and Fcγ RIII) have also been reported to mediate the MC response to other pathogens, including bacteria and parasites (32).

## MCs Innate Response to Pathogens: Release of Preformed and *De Novo* Mediators

The MC response to invading pathogens includes the release of mediators that regulate vascular flow, permeability, and cell chemotaxis. MCs can upregulate adhesion molecules on vascular endothelium, facilitating cellular recruitment from circulation into infection sites. MC-derived TNF recruits neutrophils to sites of bacterial infection (33). MC-derived eotaxin (CCL11) or IL-8 recruits eosinophils or cytotoxic cells after parasitic or viral infection, respectively (34). Eicosanoid pathways are also activated by pathogens. Leukotrienes and prostaglandins contribute to permeability, neutrophil chemotaxis and mucus production (14).

In addition, MCs can clear pathogens by releasing compounds with bactericidal activity, such as cathelicidins or β-defensins (35). Cathelicidins are host antimicrobial peptides that can be constitutively produced or induced by TLR2 signals in MCs; these peptides act in two ways: by killing pathogens and inducing MCs to migrate to the infection site (36).

## Microbe Uptake and Intracellular and Extracellular Killing

The precise role of the phagocytic capability of MCs in the host defense against bacteria remains under debate. Available evidence suggest that this role probably has a minor impact compared with the bacterial killing mediated by professional phagocytes. Recently, a review updated the knowledge about MCs phagocytic capabilities of (37). These authors compiled evidences to support the notion that several MCs membrane receptors bind free and/or opsonized pathogens. We believe that such capabilities highlight the enormous MCs versatility in controlling infectious disease during the early innate immune responses.

MCs have several mechanisms available to kill microbes they come in contact with. One of these mechanisms results from the recognition of opsonized microbes by the complement or IgG receptors. Following their binding, pathogens are internalized *via* a route involving the endosome-lysosome pathway, similar to that of phagocytes (19). However, microbe uptake can proceed without cytosolic degradation, leading to the formation of pathogen reservoirs. For most pathogens, these reservoirs have adverse effects on the host (19, 31), but other pathogens undergo intracellular degradation within MCs.

The other mechanism MCs use to kill microbes is Mast Extracellular Traps (MECT). These are antimicrobial structures that support extracellular killing of bacteria that have not been efficiently phagocytized. MECTs act through a combination of direct killing of entrapped pathogens and/or physical retention so that phagocytic cells recruited to the infection site can then eliminate the pathogen (38). These structures were first described in other cell types, including neutrophils (39).

This antimicrobial strategy implies a disruption of the nuclear membrane and a release of nuclear and granular components, which cause cell death. Some studies suggest that this process is an active and regulated event where the presence of microbes induces MECT, and the production of reactive oxygen species (ROS) leads to cell death (40).

The major components of MECTs are DNA, histones, granule proteins, such as tryptase, and antimicrobial peptides. The role of MECTs still requires further investigation; however, a possible explanation of its physiological relevance in MCs can be derived from the fact that this action is restricted to the same physical space that demonstrates MC activity. The antimicrobial action is observed locally, avoiding uncontrolled MC activation and the undesirable spread of proinflammatory mediators after an overwhelming infection to the tissue, which could lead to vascular leakage and further enhance inflammation and tissue damage (20).

## MCs in Lung Diseases

MC activity is involved in the maintenance of healthy lungs, contributing to defense against several respiratory pathogens, including *Mycobacterium tuberculosis, Staphylococcus aureus, Francisella tularensis, Streptococcus pyogenes, Klebsiella pneumoniae, Listeria monocytogenes, Pseudomona aeruginosa*, and *Streptococcus pneumoniae* (41). However, inappropriate or chronic activation of MCs by IL-9 may lead to pathologic inflammation and remodeling of tissue structures, including fibrosis during uncontrolled infections (42). There is strong evidence of MC number increase and activation in lung diseases, such as atopic and nonatopic asthma (43), while changes in the phenotypes of the MCs present in the airway (44) have been observed for pulmonary fibrosis (45) and pulmonary hypertension, which is characterized by increased pulmonary vascular resistance and remodeling (46). Mast cell activation may also play a role in acute respiratory distress syndrome (ARDS) (47) and chronic obstructive pulmonary disease (COPD) (48). Associations between MC infiltration and lung cancer prognosis give contradictory results; the differential contributions of MCs to tumor angiogenesis and tumor cytotoxicity according to the microenvironment may explain the lack of consistent conclusions (49).

SARS-CoV-2 infection in mice model have revealed the effects MCs humoral mediators. The histamine from MCs granules increases the expression of cytokines, such as IL-6, and chemokines, such as IL-8; which favor the hyperinflammatory state observed at severe stages of viral infection. The cytokines and chemokines also degrade the extracellular matrix and the hyaline membrane of the intra-alveolar space, which can lead to thrombosis (50). On the other hand, the lungs of COVID-19 patients show a diffuse alveolar damage (DAD) with suppression of MCs proliferation (51). A plausible explanation is that the high interferon levels in the early phase of COVID-19 might cause this suppression observed in DAD (51). DAD is also observed in lungs of patients ADRS, acute hypersensitivity pneumonitis, idiopathic acute interstitial pneumonia, drug abuse, toxic inhalants, ingestants, connective tissue disease, viral and bacterial infections including some acute forms of tuberculosis (52, 53). In addition, the levels of serum anti-SARS-CoV-2–spike S1 protein-specific IgE (SP-IgE) and anti-SARS-CoV-2 nucleocapsid protein-specific IgE (NP-IgE) appear to be significantly higher in patients suffering severe COVID-19; and SP-IgE levels correlated with the total lung severity scores and the ratio of arterial oxygen partial pressure to fractional inspired oxygen (PaO_2_/FiO_2_) (54). Post-acute sequalae COVID-19 patients report significantly higher expression in serum of IL-6 and CXCL1; whereas no difference was observed on serum levels of IL-8, TNF, CCL2, CCL3, IL-17A, IL-33, and VEGF (55). Taken all together these initial evidences warrant separate review to accurately compile the natural history of MCs during SARS-CoV-2 infection, disease and resolution.

## Tuberculosis

Members of the *Mycobacterium tuberculosis* complex, which include *MTB*, *M. caprae, M. microti, M. pinnipedii*, *M. africanum*, and *M. canettii* (the latter two are responsible for a small number of cases in Africa), are responsible for TB. The first three species mainly affect humans, while *M. bovis*, which is also part of the *MTB* complex group, is involved primarily in cattle infections. Mycobacteria other than tuberculosis (MOTT) can cause pulmonary TB in humans. These are also known as non-tuberculous mycobacteria (NTM) and affect mainly immunocompromised patients as well as the elderly.

The first contact between the bacilli and the patient occurs when the patient inhales microdroplets containing the bacilli, which constitutes the pulmonary transmission of TB. After this primary contact, the infection can go unnoticed for several weeks, and at this point, there are three possible outcomes. First, the infection may be controlled by the human immune cells; second, the bacilli can remain in a latent state and third, the patient may present with symptoms. Once the infection is established, granulomas, which are aggregates of infected macrophages surrounded by different immune cells, begin to form (56).

The initial MTB infection is asymptomatic. Despite symptoms initiation is variable, after approximately 4 to 6 weeks, the patient starts to present hypersensitivity to tuberculin (57). Symptoms that a patient with pulmonary TB may present include cough, fever, night sweats, weight loss, and hemoptysis.

MTB can establish either an acute infection of the lung tissue or a chronic infection where it remains inside the immune system cells. The bacteria can reside inside alveolar macrophages after entering through a phagocytotic process with the help of the complement fraction (8). However, there are other cells involved in recognizing MTB after it enters the body, as well as modulating the immune response. For example, previous studies have demonstrated that in addition to residing in macrophages, MTB infects different cell types in the lungs, including dendritic cells and neutrophils; moreover, the dominant infected cell population varies at the different stages of infection (58, 59). Pahari et al. reviewed MTB interactions using a mononuclear phagocyte system and highlighted the mechanisms involved in reversing the immune suppression induced by MTB infection (60).

TB is diagnosed by isolating and identifying MTB on sputum samples from ill patients. This can be completed by observing the bacilli under a microscope, culturing the bacilli, or by PCR (61). Other laboratory tests can also be performed, such as the tuberculin test (TST) and the Interferon Gamma test (IGRA).

For example, the TST is not completely specific, because patients that have been vaccinated with the Bacillus Calmette Guerin (BCG) vaccine will show a positive result (62). On the other hand, the IGRA tests have yielded high rates of conversions between positive and negative latent tuberculosis infection (LTBI), mainly due to the cutoff values (63). Although these tests are highly accepted, they are not foolproof and represent a challenge when trying to correctly diagnose patients. For these reasons, it is important to evaluate other methods for diagnosing patients with TB, especially patients with LTBI.

## MCs and Tuberculosis

The first evidence of MCs’ role in TB came from various studies performed over previous decades. First, Ratnam et al. studied rodent lungs after MTB infection and reported the presence of “cells containing many electron-dense intracytoplasmic granules” which were determined to be from MCs (64, 65). Later, Muñoz et al. demonstrated that MTB could activate MCs *in vitro*, resulting in MCs degranulation and production of the inflammatory cytokines TNFα and IL-6 (66). *In vivo* experimentation showed an increase in MCs after MTB infection in rodent lungs (65). Also, MC-derived TNFα may play a role in the early steps of infection since it is stored in granules and can be immediately released after infection. TNFα has an initial impact in neutrophil recruitment and leads to the differentiation of circulant monocytes to macrophages in addition to playing an important role in granuloma maintenance. An experimental animal TB model that excluded TNFα showed an increase in IL-6 production (67).

Muñoz and colleagues showed CD48 recognizes and mediates MTB uptake into MCs (66). The same authors found that MTB can also enter the MCs through lipid rafts (68). TLR2 has also been proposed to play a role in MCs after MTB infection. Transfer of TLR2^+/+^ MCs into TLR^-/-^MTB-infected mice showed diminished lung bacterial growth and an increase in proinflammatory cytokine release that contributes to granuloma maintenance (69). More recently, a study by Naqvi et al. demonstrated a massive increase in MC numbers in the infected lung of BCG infected animals (70). They also showed that *in vitro* co-culture of BCG and rodent Rat Basophilic Leukaemia (RBL-2H3) MCs led to significant killing of BCG, phagocytosis of BCG, take up BCG-derived antigens by macropinocytosis, generation of Reactive Oxygen Species (ROS) and degranulation. All together these evidences suggest that MCs play a significant protective role during tuberculosis infection and disease.

MC-mediated innate immunity usually promotes timely resolution of acute infection; however, paradoxically, this same response can also promote chronic infections and exacerbate pathologies. In the TB context, MTB uptake through raft membrane domains might also allow the bacteria to bypass the lysosome degradation route, thereby resulting in intracellular MTB survival (68). During lung mycobacterial infection, MCs are involved in the development of granulomas, which may also protect bacteria from total clearance (71). On the other hand, MTB can use additional strategies to evade the immune response; for example, MTB can produce catalase, which degrades hydrogen peroxide, thereby preventing the release of mast cells’ extracellular traps (MCETS) (72).

**Figure 1:**
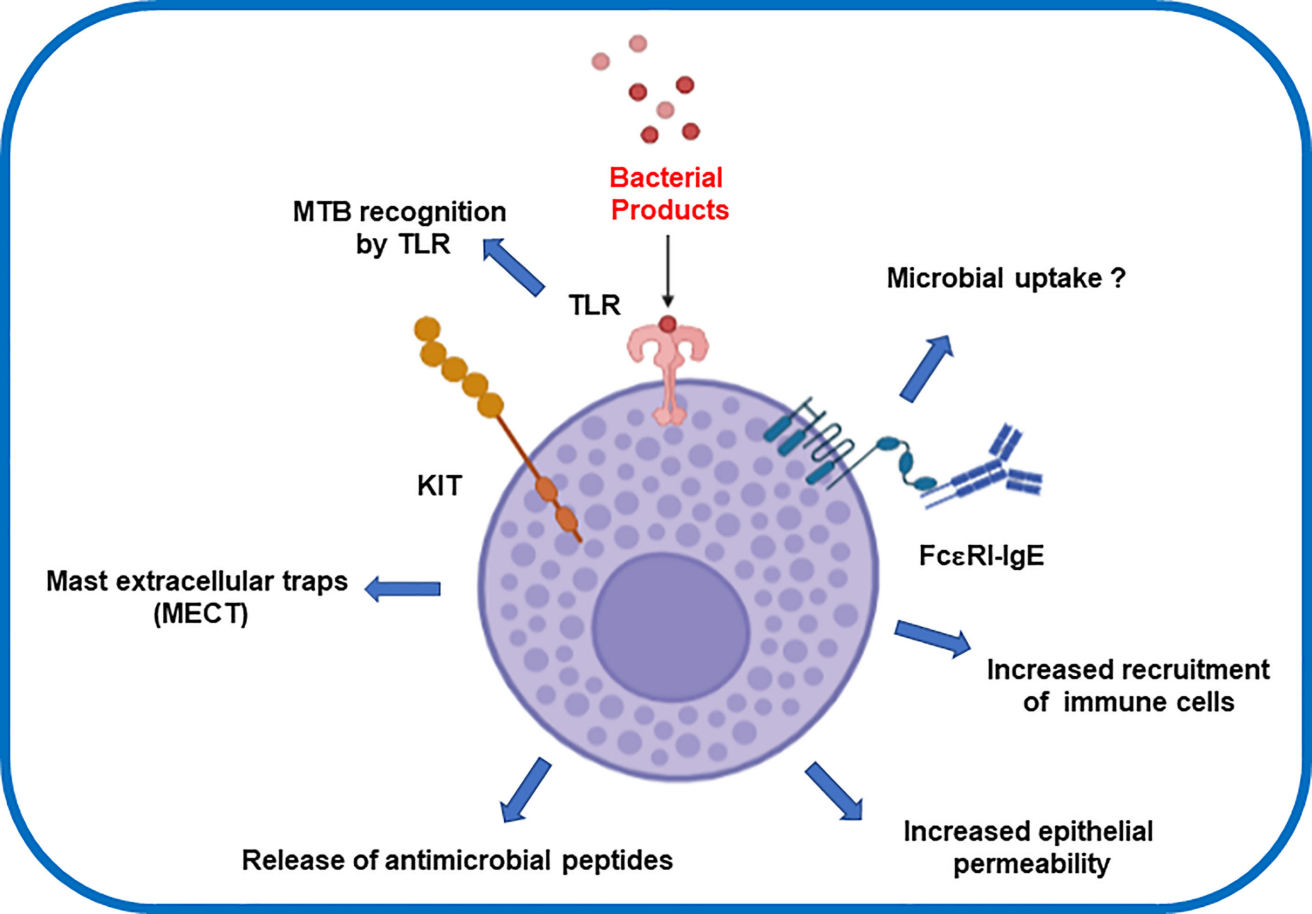
Effector inflammatory response after mast cells interaction with *Mycobacterium tuberculosis.*

Experiments using recombinant MTB antigens provide further insights into the MC response triggered by MTB. These antigens are present in a group of proteins actively produced by MTB during its growth. The recombinant MTB antigens MTSA-10, MPT-63, and ESAT-6 were found to induce the release of inflammatory mediators (66). MCs also respond specifically to MTB lipids increasing calcium influx and degranulation (73). In contrast, such immune response have been tested as therapeutic strategy for chronic diseases such as chronic spontaneous urticaria (CSU) (74). Yan and colleagues demonstrated that the use of BCG polysaccharide nucleic acid (BCG-PSN) significantly reduced the recovery time of patients suffering CSU (74). They supported this finding by showing *in-vitro* data that revealed BCG-PSN also inhibited the hexosaminidase release rates in IgE-sensitized RBL-2H3 cells. Taken all together, the modulating role of Mycobacteria promises a novel therapeutic strategy for targeting chronic autoimmune diseases. Molecular mechanisms of MCs and MTB interaction now warrant a multidisciplinary effort to generate new fundamental basis that depict the MC´s microbial intake (**Figure 1**).

The precise lipids involved and the exact MC receptors that are triggered remain unknown. Determining this is further complicated by the fact that MTB’s lipid composition varies throughout the infection process. There is a lipid profile that dampens or enhances immune responses resulting in bacterial persistence or clearance (75). This differential expression may be key in determining the clinical fate of infection. It would be key to assess MCs activation mechanisms in those scenarios. A better understanding of lipid interactions with MCs could lead to the development of immunomodulators distinct from the traditional protein antigen-based vaccines. Moreover, it would be useful to determine if MCs play a role in the establishment and perpetuation of the granuloma, as well as identify a molecule that can be used as a biomarker to classify patients in the different stages of infection.

There are also clinical evidences that support the role of MC´s in the MTB clearance during TB. An earlier study investigated the MC density on tissue sections of 45 people suffering tuberculous lymphadenitis. They found lymph node to have a significant increase of MC´s with greater granuloma involvement and multinucleated giant cell formation (76). In contrast, during tuberculous pleurisy, there is no significant increase in MC´s when compared to individuals with nonspecific pleuritis (77). When studying more severe pulmonary TB among patients admitted to the intensive care units, it was observed that these patients have an increased MCs in lung tissue (78). In fact, a recent study analyzed fibrotic tissue from post-mortem lung tissue microarrays from individuals with pulmonary TB and healthy control subjects (79). They demonstrated that MCs were localized at pneumonic areas, in the granuloma periphery and particularly abundant. Similarly, non-tuberculosis mycobacteria trigger MCs response. Emerging evidences show MCs within leprosy lesions, including collagen increase and tryptase-rich mast cell density (80, 81). Taken all together, these early clinical evidences support the notion of MCs playing a role during MTB infection and disease.

## Conclusion

The role of MCs in infectious processes, including TB infection, supports their potential use as biomarkers to predict disease severity. MCs should also be considered a therapeutic target for treating patients that do not respond as expected to anti-TB therapies. These therapies usually result in the modulation of immune cell populations, including mast cells. Without this modulation, the mycobacteria evade the immune response through the decomposition of hydrogen peroxide, which is an essential trigger for MCET induction. There is emerging *in vivo* and clinical evidences that highlight some other roles of MCs during TB. Further studies are warranted to fully characterize the complete MCs contribution not only during disease, but also during latent TB infection stage as well as clearance after anti-TB therapy.

## Author Contributions

Conceptualization: AG and IT-A; Investigation: AG, AC, MM, and IT-A; Resources: AG and MM; Writing – original draft preparation: AG, AC, MM, and IT-A; Writing – review and editing: AG, MM, and IT-A; Funding acquisition: AG, MM and IT-A. All authors contributed to the article and approved the submitted version.

## Funding

This research was partially funded by the National Secretariat of Science and Technology of Panama (SENACYT) through the Sistema Nacional de Investigadores. AG is funded by the Sistema Nacional de Investigadores SNI de Panamá de la Secretaría Nacional de Ciencia, Tecnología e Innovación (SENACYT), grant No. 22-2020. IT-A is funded by Vicerrectoría de Investigación y Postgrada, Universidad de Panamá.

## Conflict of Interest

The authors declare that the research was conducted in the absence of any commercial or financial relationships that could be construed as a potential conflict of interest.

The reviewer RMC declared a past co-authorship with the author MM to the handling editor.

## Publisher’s Note

All claims expressed in this article are solely those of the authors and do not necessarily represent those of their affiliated organizations, or those of the publisher, the editors and the reviewers. Any product that may be evaluated in this article, or claim that may be made by its manufacturer, is not guaranteed or endorsed by the publisher.
